# Cysteines have a role in conformation of the UVR8 photoreceptor

**DOI:** 10.1111/tpj.15841

**Published:** 2022-06-20

**Authors:** Xinyang Liao, Gareth I. Jenkins

**Affiliations:** ^1^ Institute of Molecular, Cell and Systems Biology, College of Medical, Veterinary and Life Sciences, Bower Building University of Glasgow Glasgow G12 8QQ UK

**Keywords:** UV‐B, UVR8, photoreceptor, photomorphogenesis, *Arabidopsis thaliana*

## Abstract

The UV RESISTANCE LOCUS 8 (UVR8) photoreceptor mediates plant responses to Ultraviolet‐B (UV‐B) wavelengths. The UVR8 dimer dissociates into monomers following UV‐B photoreception, a process accompanied by conformational changes that facilitate interaction of UVR8 with proteins that initiate responses. However, the importance of particular amino acids in maintaining UVR8 conformation and modulating protein interactions is poorly understood. Here we examine the roles of cysteine amino acids C231 and C335 in UVR8 structure and function. UVR8^C231S,C335S^ mutant protein forms dimers and monomerizes similarly to wild‐type UVR8. UVR8^C231S,C335S^ interacts with CONSTITUTIVELY PHOTOMORPHOGENIC 1 (COP1) in plants to initiate photomorphogenic responses to UV‐B, although the interaction is weaker when examined in yeast two‐hybrid assays. Similarly, the interaction of UVR8^C231S,C335S^ with REPRESSOR OF UV‐B PHOTOMORPHOGENESIS (RUP) proteins is weaker in both plants and yeast compared with wild‐type UVR8. Re‐dimerization of UVR8 in plants, which is mediated by RUP proteins, occurs with reduced efficiency in UVR8^C231S,C335S^. Fluorescence resonance energy transfer analysis indicates that UVR8^C231S,C335S^ has an altered conformation in plants, in that the N‐ and C‐termini appear closer together, which may explain the altered protein interactions.

## Significance Statement

Photomorphogenic responses to ultraviolet‐B (UV‐B) wavelengths are initiated by the UV RESISTANCE LOCUS 8 (UVR8) photoreceptor, but the importance of particular amino acids in maintaining UVR8 conformation and modulating protein interactions is poorly understood. Photobiological analyses of UVR8 mutants indicate that specific cysteine amino acids have a role in determining UVR8 conformation.

## INTRODUCTION

Ultraviolet‐B (UV‐B) wavelengths in sunlight stimulate a range of biochemical, physiological, and morphogenic responses in plants, many of which are mediated by the photoreceptor UV RESISTANCE LOCUS 8 (UVR8) (Jenkins, 2017; Podolec et al., 2021). These responses result from extensive changes in gene expression that are initiated by UVR8 photoreception and associated signalling events (Brown et al., 2005; Favory et al., 2009).

The UVR8 protein consists of a seven‐bladed β‐propeller core with flexible N‐ and C‐terminal tails (Christie et al., 2012; Wu et al., 2012). In the absence of UV‐B wavelengths, the protein forms a homodimer that is held together by salt bridges between charged amino acids on the dimer interaction surface of adjacent monomers (Christie et al., 2012; Rizzini et al., 2011; Wu et al., 2012). The photoreceptor does not have a bound cofactor as its chromophore, and photoreception involves the absorption of UV‐B and short wavelength UV‐A light by tryptophan (Trp) amino acids in the primary sequence. UVR8 has 14 Trps, seven of which are in the dimer interaction surface of each monomer (Christie et al., 2012; O'Hara & Jenkins, 2012; Wu et al., 2012). Of these seven, W233, W285, and W337 form an excitonically coupled cluster, together with W94 on the adjacent monomer. Excitation energy is transferred from multiple Trps to W233 and W285 in the dimer interaction surface (Li et al., 2020). Excitation of these Trps, which are adjacent to key charged amino acids, results in neutralization of salt bridges between monomers and hence dissociation of the dimer (Mathes et al., 2015).

Following UV‐B exposure of plants, UVR8 accumulates in the nucleus, where it regulates gene expression (Kaiserli & Jenkins, 2007). UVR8 monomers initiate signal transduction processes through interaction with other proteins. Interaction of monomeric UVR8 with CONSTITUTIVELY PHOTOMORPHOGENIC 1 (COP1), a substrate acceptor for an E3 ubiquitin ligase complex, allows accumulation of the ELONGATED HYPOCOTYL 5 (HY5) transcription factor (Favory et al., 2009; Huang et al., 2013), which is a key positive regulator of responses mediated by UVR8 (Brown et al., 2005; Brown & Jenkins, 2008; Favory et al., 2009). UVR8 is also able to interact with several transcription factors, WRKY36 (Yang et al., 2018), BIM1 and BES1 (Liang et al., 2018), MYB73/MYB77 (Yang et al., 2020) and MYB13 (Qian et al., 2020), which modulates their ability to regulate particular sets of genes, leading to specific downstream responses. In addition, monomeric UVR8 interacts with REPRESSOR OF UV‐B PHOTOMORPHOGENESIS (RUP) proteins, RUP1 and RUP2, which promote re‐dimerization of UVR8 (Heijde & Ulm, 2013; Liao et al., 2020). RUP proteins bind to a short region within the C‐terminus, termed the C27 region (Cloix et al., 2012), and impair binding of COP1 to C27 (Heijde & Ulm, 2013). Thus, RUP proteins act as negative regulators of UVR8 signalling (Gruber et al., 2010).

Site‐specific mutagenesis combined either with examination of mutant UVR8 proteins *in vitro* or with functional studies *in vivo* has determined the roles of specific amino acids in UVR8 structure and function, notably charged amino acids that maintain the dimer, specific Trps involved in photoreception, and amino acids that are critical for interactions with other proteins (Jenkins, 2014; Podolec et al., 2021). Nevertheless, there is much to be learnt about the structure–function relationships of UVR8. The focus of the present study is on the cysteine amino acids of UVR8. Cysteines are important in protein secondary structure, through formation of disulphide bonds, and in a variety of post‐translational modifications. There is no evidence from crystal structures that disulphide bonds are present in dimeric UVR8 (Christie et al., 2012; Wu et al., 2012), and treatment of the protein with strong oxidizing or reducing agents does not affect monomer formation (Wu et al., 2012). However, it has been suggested that oxidation of a reduced amino acid, potentially a cysteine, could enhance structural flexibility of the protein and facilitate re‐dimerization (Miyamori et al., 2015). Hence, in this study we used mutagenesis to investigate the potential importance of specific cysteines in UVR8 structure and function.

## RESULTS

### Location of UVR8 cysteines

**Figure 1:**
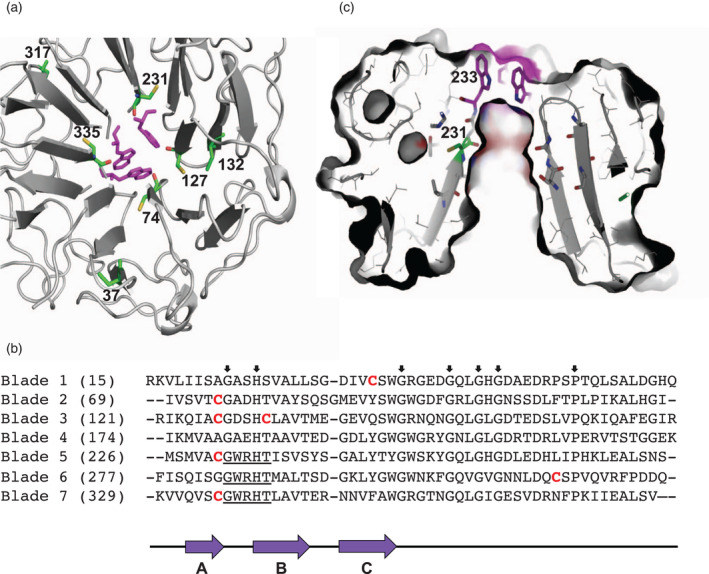
Position of UVR8 cysteines. (a) UVR8 monomer looking towards the dimer interaction surface. Seven cysteines are shown in green and numbered. Trps W233, W285, and W337 are shown in magenta. Image made using Pymol. (b) Position of cysteines (in red) in the β‐propeller blades of UVR8. Sequences of the blades are aligned using invariant amino acids (arrowed above sequences). The first amino acid of each blade is numbered (in brackets). The GWRHT motif containing W233, W285, and W337 is underlined. Three regions of β‐strand, A, B, and C, are indicted below the sequences by purple arrows; the black lines represent loops associated with each blade. (c) Section through the UVR8 monomer viewed from the side showing the water‐filled depression below the dimer interaction surface. Trps W233, W285 and W337 are shown in magenta. C231 is shown in green, adjacent to the depression and below W233. Image made using Pymol. [Colour figure can be viewed at wileyonlinelibrary.com]

UVR8 has seven cysteines (Figure 1a,b), but their functional significance is unknown. C37 is in blade 1 of the β‐propeller; C74 is in blade 2; C127 and C132 are on adjacent β‐strands of blade 3; C231 is in blade 5; C335 is in blade 7; and C317 is in a loop between blades 6 and 7 and is surface exposed. C74, C127, C231, and C335 are in conserved locations at the end of the first β‐strand of blades 2, 3, 5, and 7 respectively, and are adjacent to the water‐filled depression in the centre of the β‐propeller that extends below the dimer interface (Figure 1c). It is intriguing that C231 and C335 are located just below the interface Trps W233 and W337. Moreover, the positions of C231 and C335 in a CGWRHT motif are conserved in UVR8 sequences from diverse angiosperms, the lycopodiophyte *Selaginella*, bryophytes, and green algae (Figure S1). We therefore focused this study on C231 and C335.

### **UVR8** ^**C231S**^ ^**,C335S**^ **presents normal dimer/monomer formation and UV‐B photoreception**

**Figure 2:**
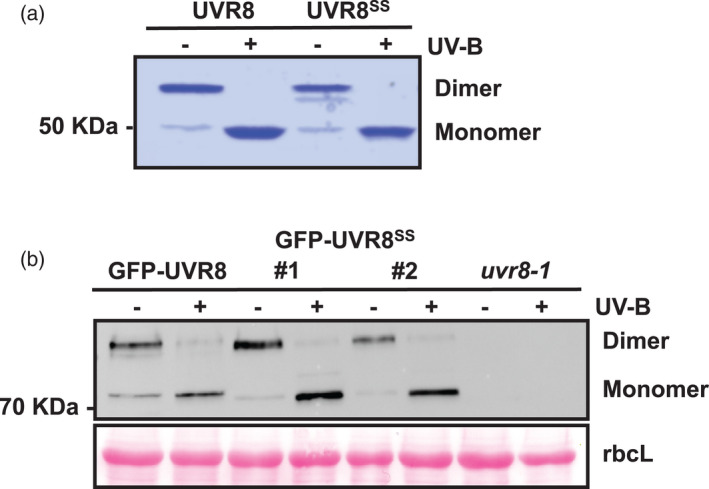
Dimer‐monomer status of UVR8^C231S,C335S^ protein *in vitro* and *in vivo*. (a) Purified UVR8 and UVR8^C231S,C335S^ (UVR8^SS^) proteins were resolved by sodium dodecyl sulphate–polyacrylamide gel electrophoresis with non‐boiled samples and stained with Coomassie brilliant blue after being exposed (+) or not (−) to 3 μmol m^−2^ sec^−1^ broadband UV‐B for 10 min. (b) *uvr8‐1/35Spro:GFP‐UVR8* (GFP‐UVR8)*, uvr8‐1/35Spro:GFP‐UVR8* ^*C231S,C335S*^ (GFP‐UVR8^SS^; two independent lines), and *uvr8‐1* plants grown in 120 μmol m^−2^ sec^−1^ white light for 14 days were exposed (+) or not (−) to 3 μmol m^−2^ sec^−1^ broadband UV‐B for 1 h. Protein extracts were separated by sodium dodecyl sulphate–polyacrylamide gel electrophoresis with non‐boiled samples and the immunoblot was probed with an anti‐UVR8‐C antibody. Ponceau S‐stained Rubisco large subunit (rbcL) bands are shown as a loading control. [Colour figure can be viewed at wileyonlinelibrary.com]

To examine the potential importance of C231 and C335 in UVR8 structure and function, site‐specific mutagenesis was used to convert both cysteines to serine, which is very similar in structure to cysteine. To test whether the C231S and C335S mutations affect the stability of the UVR8 homodimer and UV‐B‐induced monomerization, UVR8^C231S,C335S^ (referred to in figures as UVR8^SS^) was expressed in *Escherichia coli* and purified. The protein was examined using sodium dodecyl sulphate–polyacrylamide gel electrophoresis (SDS‐PAGE) with unboiled samples, which has been used extensively to test dimer/monomer status. Similar to wild‐type UVR8, the UVR8^C231S,C335S^ protein presented as a dimer before UV‐B exposure and responded to UV‐B exposure by converting to the monomer (Figure 2a).

To examine dimer/monomer status and function in plants, we generated transgenic lines expressing UVR8^C231S,C335S^ as a green fluorescent protein (GFP) fusion in the *uvr8‐1* null mutant background. The expression level of GFP‐UVR8^C231S,C335S^ in plants was similar to that of the previously generated GFP‐UVR8 line (Figure 2b). Consistent with the purified protein, GFP‐UVR8^C231S,C335S^ extracted from seedlings was predominantly present as a homodimer in the absence of UV‐B and as a monomer after UV‐B exposure (Figure 2b). These results clearly demonstrate that the C231S and C335S mutations of UVR8 do not impair dimer formation and UV‐B‐induced monomerization.

### **GFP‐UVR8** ^**C231S**^ ^**,C335S**^ **is functional in initiating UV‐B‐induced photomorphogenic responses in plants**

**Figure 3:**
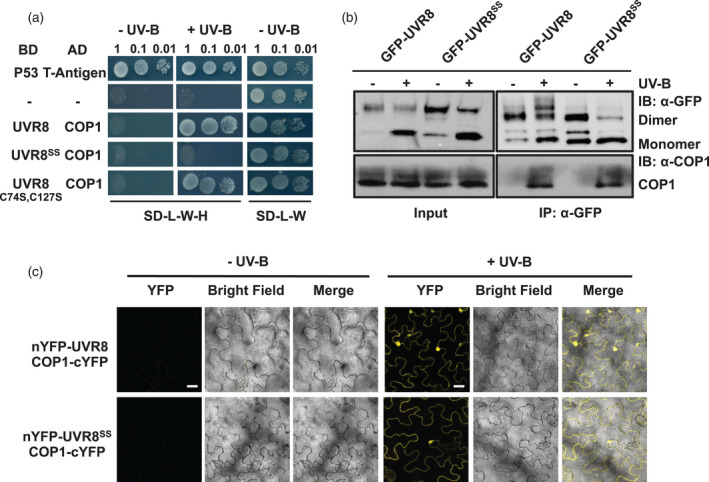
Interaction of UVR8^C231S,C335S^ with COP1 in plants and in yeast two‐hybrid assays. (a) Yeast two‐hybrid assays of COP1 interactions with wild‐type UVR8, UVR8^C231S,C335S^ (UVR8^SS^), and UVR8^C74S,C127S^. Suspended cells in serial dilutions (OD = 1, 0.1, 0.01) were spotted on selective medium (SD/−Leu/−Trp/–His, SD‐L‐W‐H) or non‐selective medium (SD/−Leu/−Trp, SD‐L‐W). Growth was assayed in the presence (+UV‐B) or absence (−UV‐B) of UV‐B. AD, activation domain; BD, binding domain. P53 and T‐antigen were used as a positive control and empty vectors (−) as a negative control. (b) Co‐immunoprecipitation assay of COP1 interactions with wild‐type GFP‐UVR8 and GFP‐UVR8^C231S,C335S^. Protein extracts from 14‐day‐old plants expressing *uvr8‐1/35Spro:GFP‐UVR8* (GFP‐UVR8) or *uvr8‐1/35Spro:GFP‐UVR8* ^*C231S,C335S*^ (GFP‐UVR8^SS^) were treated with 3 μmol m^−2^ sec^−1^ broadband UV‐B (+) or left in white light (−) for 15 min. Co‐immunoprecipitation assays of extracts were performed with anti‐GFP antibody under the same light conditions. Input samples (15 μg, input) and eluates (IP) were resolved on sodium dodecyl sulphate–polyacrylamide gel electrophoresis with non‐boiled samples and immunoblots were probed with anti‐GFP and anti‐COP1 antibodies. IB, immunoblot; IP, immunoprecipitation. (c) BiFC assays of COP1 interactions with wild‐type UVR8 and UVR8^C231S,C335S^. Plasmids expressing N‐terminal‐YFP fused to either UVR8 or UVR8^C231S,C335S^ (UVR8^SS^) together with C‐terminal‐YFP fused to COP1 were infiltrated into *Nicotiana benthamiana* leaves. Plants were exposed (+UV‐B) or not (−UV‐B) to 3 μmol m^−2^ sec^−1^ broadband UV‐B for 1 h before taking images. Images from left to right are YFP fluorescence signal, bright field, and merged image. Bar = 20 μm. Negative control transformations are shown in Figure S3. [Colour figure can be viewed at wileyonlinelibrary.com]

Interaction of UVR8 monomers with COP1 is required for the photoreceptor to initiate responses. We therefore tested whether the cysteine mutations affect this interaction and UVR8‐mediated responses. Yeast two‐hybrid (Y2H) assays indicated that UVR8^C231S,C335S^ interacts much less strongly with COP1 than wild‐type UVR8 (Figure 3a), whereas the single UVR8^C231S^ and UVR8^C335S^ mutants appeared similar to wild‐type (Figure S2a). As a control, we examined interaction in yeast between COP1 and UVR8 mutated in the other two cysteines adjacent to the water‐filled depression, C74 and C127; interaction of COP1 with UVR8^C74S,C127S^ was similar to wild‐type. With respect to plants, co‐immunoprecipitation (Co‐IP) assays in *Arabidopsis* transgenic lines (Figure 3b) and bimolecular fluorescence complementation (BiFC) assays in *Nicotiana benthamiana* leaves (Figures 3c and S3) revealed that UVR8^C231S,C335S^ interacts with COP1 in plants in the presence of UV‐B, similar to the wild‐type.

**Figure 4:**
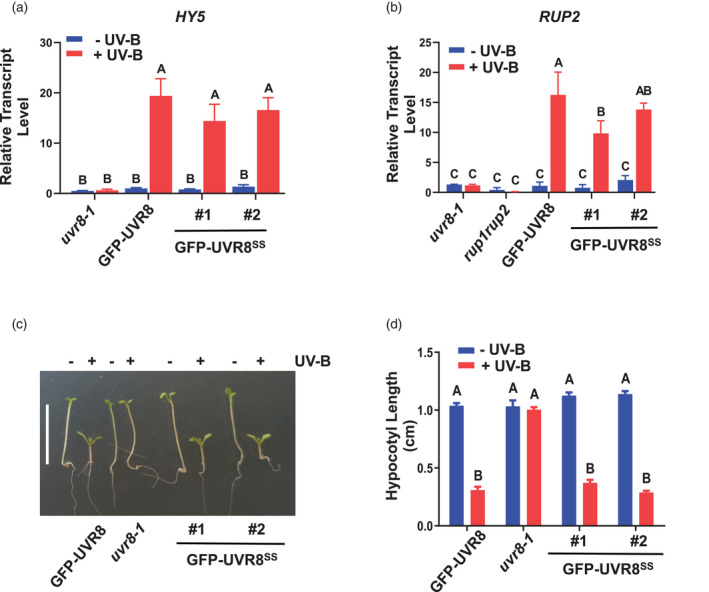
GFP‐UVR8^C231S,C335S^ mediates photomorphogenic responses to UV‐B. (a) *HY5* and (b) *RUP2* transcript levels were measured by reverse transcription–quantitative polymerase chain reaction. *uvr8‐1/35Spro:GFP‐UVR8* (GFP‐UVR8)*, uvr8‐1/35Spro:GFP‐UVR8* ^*C231S,C335S*^ (GFP‐UVR8^SS^; two independent lines) and *uvr8‐1* plants were grown under 120 μmol m^−2^ sec^−1^ white light for 14 days and exposed (+UV‐B) or not (−UV‐B) to 3 μmol m^−2^ sec^−1^ broadband UV‐B for 3 h before harvest. Data are mean ± SE, *n* = 3 biological replicates; letters indicate statistically significant mean values (*P* < 0.01; two‐way anova with Tukey's test). (c) Hypocotyl elongation assay. Plants were grown under 1.5 μmol m^−2^ sec^−1^ white light with (+UV‐B) or without (−UV‐B) supplementary 1.5 μmol m^−2^ sec^−1^ narrowband UV‐B for 5 days. Bar = 1 cm. (d) Quantification of hypocotyl length as in (c). Mean ± SE, *n* = 25; letters indicate statistically significant mean values (*P* < 0.01; two‐way anova with Tukey's test). [Colour figure can be viewed at wileyonlinelibrary.com]

We analysed two UV‐B responses in GFP‐UVR8^C231S,C335S^ transgenic lines. First, quantitative reverse transcription‐polymerase chain reaction (PCR) analysis showed that the transcript levels of *HY5* and *RUP2*, which are well known genes induced by UV‐B, were upregulated after UV‐B exposure in both GFP‐UVR8 and GFP‐UVR8^C231S,C335S^ lines (Figure 4a,b). Secondly, a hypocotyl elongation assay revealed that the GFP‐UVR8^C231S,C335S^ lines displayed a comparable photomorphogenic growth suppression response to the GFP‐UVR8 plants in the presence of UV‐B, and a normal long hypocotyl phenotype in the absence of UV‐B (Figure 4c,d). Taken together, these results demonstrate that GFP‐UVR8^C231S,C335S^ presents UV‐B‐induced photomorphogenesis and rescues the *uvr8‐1* phenotype, similar to wild‐type GFP‐UVR8.

### Binding affinity of UVR8^C231S,C335S^ for RUP2 and negative regulation of UVR8 signalling are partly impaired

**Figure 5:**
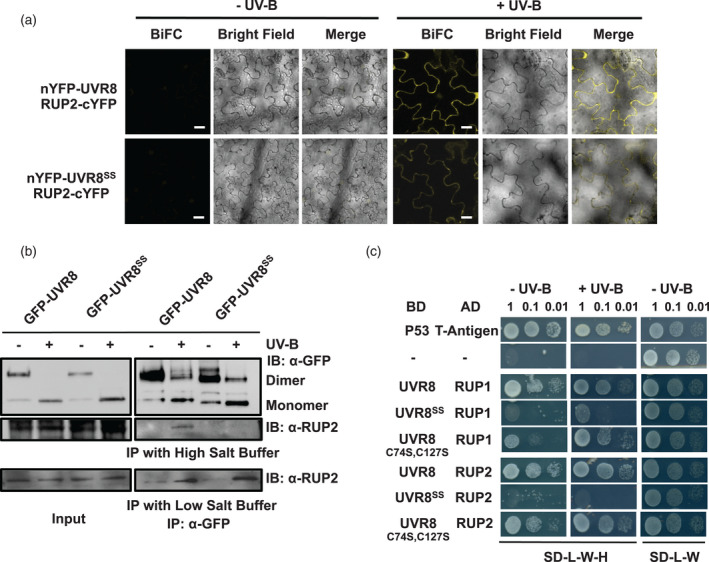
C231S and C335S partially weaken the interaction between UVR8 and RUP2. (a) BiFC assays of RUP2 interactions with wild‐type UVR8 and UVR8^C231S,C335S^. Plasmids expressing N‐terminal‐YFP fused to either UVR8 or UVR8^C231S,C335S^ (UVR8^SS^) together with C‐terminal‐YFP fused to RUP2 were infiltrated into *Nicotiana benthamiana* leaves. Plants were exposed (+UV‐B) or not (−UV‐B) to 3 μmol m^−2^ sec^−1^ broadband UV‐B for 1 h before taking images. Images from left to right are YFP fluorescence signal, bright field, and merged image. Bar = 20 μm. Negative control transformations are shown in Figure S3. (b) Co‐immunoprecipitation assay of RUP2 interactions with wild‐type GFP‐UVR8 and GFP‐UVR8^C231S,C335S^. Protein extracts from 14‐day‐old plants expressing *uvr8‐1/35Spro:GFP‐UVR8* (GFP‐UVR8) or *uvr8‐1/35Spro:GFP‐UVR8* ^*C231S,C335S*^ (GFP‐UVR8^SS^) were treated with 3 μmol m^−2^ sec^−1^ broadband UV‐B (+) or left in white light (−) for 15 min. Co‐immunoprecipitation assays of extracts were performed with anti‐GFP antibody under the same light conditions. The wash buffer contained either 450 mm NaCl (high salt) or 150 mm NaCl (low salt). Input samples (15 μg, input) and eluates (IP) were resolved on sodium dodecyl sulphate–polyacrylamide gel electrophoresis with non‐boiled samples and immunoblots were probed with anti‐GFP and anti‐RUP2 antibodies. (c) Yeast two‐hybrid assays of RUP1 and RUP2 interactions with wild‐type UVR8, UVR8^C231S,C335S^ (UVR8^SS^) and UVR8^C74S,C127S^. Suspended cells in serial dilutions (OD = 1, 0.1, 0.01) were spotted on selective medium (SD/−Leu/−Trp/–His, SD‐L‐W‐H) or non‐selective medium (SD/−Leu/−Trp, SD‐L‐W). Growth was assayed in the presence (+UV‐B) or absence (−UV‐B) of UV‐B. P53 and T‐antigen were used as a positive control and empty vectors (−) as a negative control. AD, activation domain; BD, binding domain; BiFC, bimolecular fluorescence complementation; IB, immunoblot; IP, immunoprecipitation. [Colour figure can be viewed at wileyonlinelibrary.com]

UVR8^C231S,C335S^ was shown to interact with RUP2 in plants using the BiFC (Figures 5a and S3) and Co‐IP assays (Figure 5b). However, it should be noted that the interaction between GFP‐UVR8^C231S,C335S^ and RUP2 was only detected by Co‐IP under reduced stringency conditions with low salt buffer, whereas no signal was observed if the Co‐IP assay was performed with high salt buffer, indicating that GFP‐UVR8^C231S,C335S^ and RUP2 have a reduced interaction in plants (Figure 5b). Similarly, the Y2H assay revealed that the interaction of UVR8^C231S,C335S^ with RUP1 and RUP2 was impaired in yeast (Figure 5c), whereas interaction of the RUPs with UVR8^C74S,C127S^ was similar to wild‐type. A reduced interaction was also seen with the single UVR8^C231S^ and UVR8^C335S^ mutants (Figure S2b).

**Figure 6:**
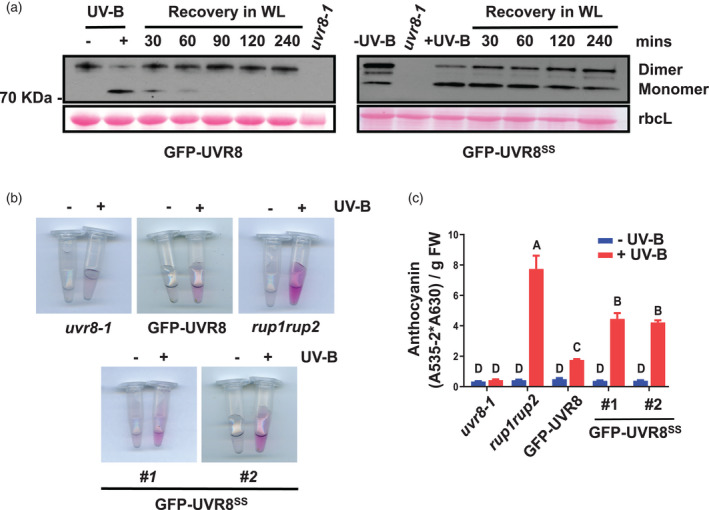
Plants expressing GFP‐UVR8^C231S,C335S^ have impaired re‐dimerization of UVR8 and enhanced anthocyanin accumulation. (a) In *vivo* re‐dimerization assay of GFP‐UVR8 and GFP‐UVR8^C231S,C335S^. Plants expressing *uvr8‐1/35Spro:GFP‐UVR8* (GFP‐UVR8) or *uvr8‐1/35Spro:GFP‐UVR8* ^*C231S,C335S*^ (GFP‐UVR8^SS^) were grown under 120 μmol m^−2^ sec^−1^ white light for 14 days and were irradiated (+UV‐B) with supplementary 3 μmol m^−2^ sec^−1^ broadband UV‐B for 30 min, then returned to white light (WL) for the indicated times. Protein extracts were resolved by sodium dodecyl sulphate–polyacrylamide gel electrophoresis with non‐boiled samples for the immunoblot analysis. The UVR8 protein was probed with the anti‐UVR8‐C antibody. Protein extracts from *uvr8‐1* plants were used as a negative control. Ponceau S stained Rubisco large subunit (rbcL) bands are shown as a loading control. (b) Anthocyanin extracts of equal numbers of seedlings grown under 1.5 μmol m^−2^ sec^−1^ white light with (+UV‐B) or without (−UV‐B) supplementary 1.5 μmol m^−2^ sec^−1^ narrowband UV‐B for 5 days. (c) Anthocyanin content was calculated as (A535‐2*A630)/fresh weight (g). Mean ± SE, *n* = 3 biological replicates; letters indicate statistically significant mean values (*P* < 0.01; two‐way anova with Tukey's test). [Colour figure can be viewed at wileyonlinelibrary.com]

The RUP interaction promotes the re‐dimerization of UVR8 monomers and negatively regulates UVR8 signalling (Gruber et al., 2010; Heijde et al., 2013). Therefore, we examined whether the weakened RUP2 binding had any effect on UVR8 negative regulation. Compared with GFP‐UVR8, the re‐dimerization of GFP‐UVR8^C231S,C335S^ monomers following transfer of UV‐B‐exposed plants to minus‐UV‐B conditions was significantly inhibited *in vivo* (Figure 6a). In addition, GFP‐UVR8^C231S,C335S^ seedlings accumulated more anthocyanin under UV‐B than GFP‐UVR8 seedlings, whereas there is no significant difference in the absence of UV‐B; however, the magnitude of increase in anthocyanin was less than in the *rup1rup2* mutant (Figure 6b,c). Taken together, these results indicate that the C231S and C335S mutations reduce the strength of interaction between UVR8^C231S,C335S^ and RUP proteins, resulting in impaired UVR8 re‐dimerization and weakened negative regulation of UVR8 signalling.

### UVR8^C231S,C335S^ has an altered conformation

**Figure 7:**
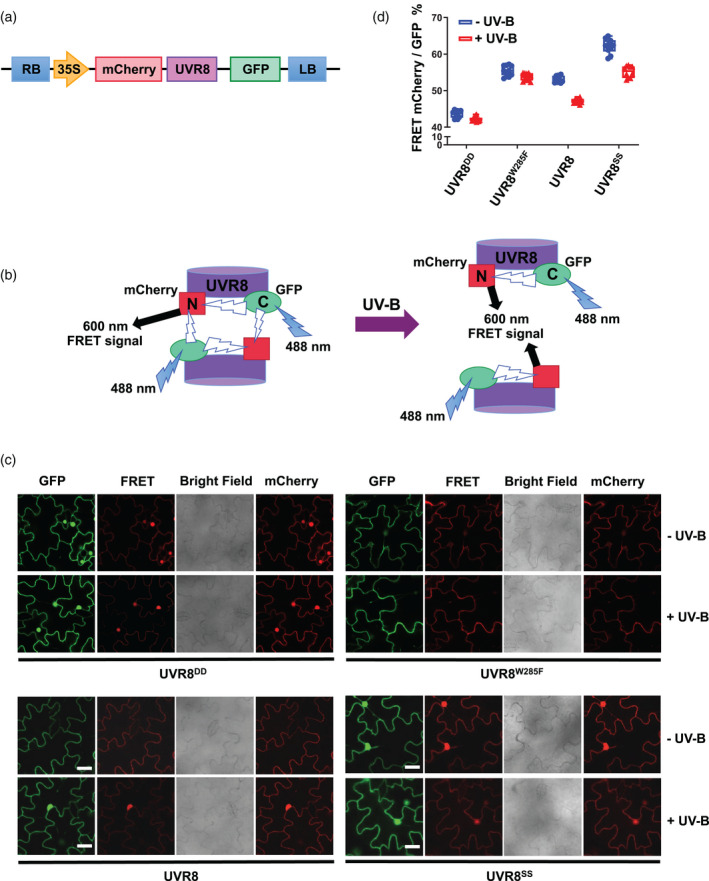
Mutations at Cys231 and Cys335 cause a conformational change of the UVR8 protein. (a) Schematic diagram of the construct of UVR8 inserted into the pFRET‐NcCg‐DEST vector. (b) GFP is excited at 488 nm and may excite mCherry causing fluorescence at 600 nm, which is the FRET signal. FRET efficiency will depend on the distance between GFP and mCherry. FRET may originate from intermolecular or intramolecular sources. Modified from Liao et al. (2019). (c) FRET assay of UVR8, UVR8^D96N,D107N^ (UVR8^DD^), UVR8^W285F^, and UVR8^C231S,C335S^ (UVR8^SS^). Images were captured from tobacco leaves transiently expressing the fusions in the pFRET‐NcCg‐DEST vector. Images show fluorescence from GFP (488 nm excitation, 500–535 nm emission) or mCherry (552 nm excitation, 590–645 nm emission), the FRET signal (488 nm excitation, 590–645 nm emission) and bright field. Plants were exposed (+UV‐B) or not (−UV‐B) to 3 μmol m^−2^ sec^−1^ broadband UV‐B for 1 h before taking images. Bar = 50 μm. (d) Quantification of FRET efficiency from images shown as in (c). For each experiment, data were collected from 10 images, and 20 regions of each image were selected at random to calculate the mean fluorescence intensity. The FRET efficiency was calculated as the mean fluorescence intensity mCherry (488 excitation)/GFP (488 excitation) and presented as a percentage. Means ± SE, *n* = 10. FRET, fluorescence resonance energy transfer. [Colour figure can be viewed at wileyonlinelibrary.com]

Previous research demonstrated that RUPs bind specifically to the C27 domain of UVR8, in contrast to COP1, which interacts with UVR8 via both the C27 domain and UVR8 core domain (Cloix et al., 2012; Yin et al., 2015). To explain why mutations of C231 and C335, which are located in the core domain of UVR8, impair interaction with RUPs, we hypothesized that these mutations might cause a conformational change of UVR8, affecting the accessibility of the C‐terminus to binding by RUPs. To investigate this notion, we analysed the conformation of UVR8^C231S,C335S^ using a fluorescence resonance energy transfer (FRET)‐based method we reported previously (Liao et al., 2019). As shown in Figure 7a, the pFRET‐NcCg‐DEST vector contains FRET‐pair tags mCherry and GFP, which are fused to the N‐ and C‐termini of the target protein (UVR8), respectively. The excited GFP can excite mCherry through FRET if they are sufficiently close, and the efficiency of FRET is proportional to the relative distance between GFP and mCherry. Therefore, both monomerization and conformational change of UVR8 may cause a change in the FRET efficiency (Figure 7b).

Wild‐type UVR8 and UVR8^C231S,C335S^ were inserted into the pFRET‐NcCg‐DEST vector. The constructs expressing the constitutively monomeric mutant UVR8^D96N,D107N^ (UVR8^DD^) and constitutively dimeric mutant UVR8^W285F^ were created as controls. Regardless of the presence or absence of UV‐B, the FRET efficiency of UVR8^W285F^ is consistently relatively high, while that of UVR8^D96N,D107N^ is consistently relatively low (Figure 7c,d), similar to the previous findings (Liao et al., 2019), indicating that much of the FRET signal is generated between adjacent monomers (Figure 7b). The FRET efficiency of UVR8 and UVR8^C231S,C335S^ dramatically reduced following UV‐B exposure (Figure 7c,d), which is explained by the monomerization of UVR8 dimers eliminating FRET between adjacent monomers (Figure 7b; Liao et al., 2019). Excluding the structural changes caused by UVR8 monomerization, the FRET efficiencies of both UVR8^C231S,C335S^ dimer and monomer were found to be much higher than those of UVR8 dimer and monomer, respectively, suggesting that the N‐ and C‐termini are closer together in both UVR8^C231S,C335S^ dimer and monomer, indicating an altered conformation (Figure 7c,d).

## DISCUSSION

The structure of UVR8 is known to be critical to its function, with the formation of monomers essential to initiate signalling (Rizzini et al., 2011). Moreover, several studies report that UVR8 undergoes major conformational changes following photoreception (Camacho et al., 2019; Heilmann et al., 2014; Miyamori et al., 2015; Zeng et al., 2015). Both dynamic X‐ray crystallography (Zeng et al., 2015) and native mass spectrometry (Camacho et al., 2019) with purified UVR8 provide evidence of partial unravelling of the β‐propeller core during monomer formation. The formation of an extended monomer structure (Camacho et al., 2019) is believed to facilitate interactions with other proteins by modifying accessibility of the C‐terminal domain. However, the processes involved in generating conformational changes in UVR8 are unknown. Cysteines are important in determining protein structure, but the available crystal structures provide no evidence of disulphide bond formation in the UVR8 dimer. However, it is unknown whether such bonds could form, and potentially break, during the proposed extensive remodelling of UVR8 structure following photoreception. It has been suggested that oxidation of a cysteine might promote re‐dimerization (Miyamori et al., 2015), but direct evidence is lacking. The data presented here are valuable, as they suggest that C231 and C335 influence the conformation of UVR8 and affect interactions with other proteins.

The FRET assay was developed previously to examine the effect of UV‐B exposure on UVR8 conformation in plants (Liao et al., 2019). The design of the pFRET‐NcCg‐DEST vector constructs enables both inter‐molecular and intra‐molecular changes to be observed by measuring the efficiency of FRET. The conversion of dimer to monomer in wild‐type UVR8 was found to cause a large reduction in the FRET efficiency, consistent with the data obtained for control mutant proteins that are constitutively either dimeric (O'Hara & Jenkins, 2012; Rizzini et al., 2011) or monomeric (Heilmann et al., 2016). The UVR8^C231S,C335S^ mutant protein has a higher FRET efficiency than wild‐type UVR8 in both the dimer and the monomer forms, indicating that in both cases the N‐ and C‐termini are closer together than in the wild‐type. Hence, the FRET experiments provide evidence that C231 and C335 have a role in maintaining the conformation of UVR8, although the underlying molecular basis is not clear.

The UVR8^C231S,C335S^ mutant protein is able to form a dimer and carry out photoreception to generate monomers. However, there is evidence of altered interaction with both COP1 and RUP proteins. The UV‐B‐dependent interaction with COP1 appears normal in plants, but is weaker in yeast, based on the Y2H assays. Under the strong selection used in these assays the interaction of COP1 and UVR8^C231S,C335S^ is clearly impaired compared with wild‐type UVR8 and the control UVR8^C74S,C127S^ fusion. Similarly, interaction of both RUP1 and RUP2 with UVR8^C231S,C335S^ is weaker in the Y2H assay. The RUP2 interaction is also weaker in plants, as shown when stringent conditions are used in the Co‐IP assay. RUP1 is expressed at very low levels in plants (Liao et al., 2020), so the extent of its interaction with UVR8 could not be assayed *in vivo*. Together the yeast and plant data indicate that UVR8^C231S,C335S^ has a reduced affinity for binding COP1 and RUPs. One possible explanation is that the altered conformation of the mutant influences the accessibility of the C27 region to the interacting proteins. The greater impact on RUP binding, at least in plants, may be because RUP proteins interact exclusively with C27 whereas COP1 also binds to the β‐propeller core.

The GFP‐UVR8^C231S,C335S^ mutant is able to initiate photomorphogenic responses in plants, similarly to wild‐type. This is not surprising as these responses are dependent on the interaction of UVR8 with COP1, which appears largely unaffected in plants. The UVR8‐COP1 interaction following UV‐B exposure leads to the accumulation of HY5 protein and the stimulation of *HY5* transcript accumulation, the latter of which was evident in GFP‐UVR8^C231S,C335S^. The interaction of UVR8 with RUP proteins displaces COP1 and promotes re‐dimerization of monomers, hence negatively regulating UVR8 activity (Heijde et al., 2013). The observed weaker interaction of GFP‐UVR8^C231S,C335S^ with RUPs most likely explains its reduced efficiency of re‐dimerization. *Arabidopsis rup1,rup2* mutant plants are impaired in re‐dimerization (Findlay & Jenkins, 2016; Heijde et al., 2013) and display a phenotype of enhanced photomorphogenic responses (Gruber et al., 2010). The GFP‐UVR8^C231S,C335S^ mutant exhibits an increase in anthocyanin formation, although not to the extent seen in the *rup1,rup2* mutant. No evidence of a hyper‐response was observed for the transcript accumulation and hypocotyl growth suppression responses examined, and the reason for this difference is not clear. UVR8 is known to interact with multiple proteins involved in various UV‐B responses, so it is possible that the altered conformation of UVR8^C231S,C335S^ also affects the binding of a transcription factor specifically involved in anthocyanin formation.

In conclusion, much remains to be understood about the structural basis of UVR8 function. In particular, little is known about the roles of specific amino acids in controlling the dynamic changes in UVR8 conformation that follow photoreception, which are crucial in determining interactions with proteins involved in effecting downstream responses and coordinating the dimer/monomer photo‐equilibrium. The data presented here indicate that specific cysteines modulate UVR8 conformation and consequently affect protein interactions, and hence further investigation of their roles is merited, for example by using native mass spectrometry (Camacho et al., 2019). As UVR8 mediates many important responses in plants, including those that affect yield and nutritional quality, understanding the molecular basis of its activity has potential applications in crop improvement.

## EXPERIMENTAL PROCEDURES

### Plant material and growth conditions

Wild‐type *Arabidopsis thaliana* (L*er*) was obtained from the European *Arabidopsis* Stock Centre (NASC, Nottingham, UK). The *uvr8‐1* and *rup1 rup2* mutant plants were reported previously (Findlay & Jenkins, 2016; Kliebenstein et al., 2002). The *uvr8‐1/CaMV35Spro:GFP‐UVR8* transgenic line was described by Cloix and Jenkins (2008). The GFP‐UVR8^C231S,C335S^ mutant was generated by site‐directed mutagenesis and cloned into the pEZR(K)L‐C vector using the methods of Cloix et al. (2012). All primers used in this study are listed in Table S1. The verified construct containing GFP‐UVR8^C231S,C335S^ was introduced into *uvr8‐1* mutant plants to generate the *uvr8‐1/CaMV35Spro:GFP‐UVR8* ^*C231S,C335S*^ transgenic line.

Plants were grown on agar plates containing half‐strength Murashige and Skoog salts in a growth cabinet at 20°C. Plants were grown for 2 weeks in a 16‐h white light/8‐h dark photoperiod, with 120 μmol m^−2^ sec^−1^ white light provided by warm‐white LEDs. For treatment with UV‐B, plants were exposed to 3 μmol m^−2^ sec^−1^ broadband UV‐B from UV‐B‐313 fluorescent tubes (Q‐Lab Co., Cleveland, OH, USA) covered by cellulose acetate film to filter out UV‐C. The spectrum of the UV‐B source was reported by Cloix et al. (2012). Fluence rates were measured with a Skye Spectrosense 1 meter (Skye Instruments, Llandrindod Wells, Wales) with a SKU 430 sensor (Skye Instruments).

### Photomorphogenic responses

Quantitative reverse transcription‐PCR assays of *HY5* and *RUP2* transcripts in RNA samples isolated from leaf tissue were undertaken exactly as described by Díaz‐Ramos et al. (2018), with transcript levels quantified relative to *ACTIN2* control transcripts. The primers are listed in Table S1.

The hypocotyl elongation assay was performed as described by Cloix et al. (2012). Plants were grown for 4 days in 1.5 μmol m^−2^ sec^−1^ white light with or without 1.5 μmol m^−2^ sec^−1^ narrowband UV‐B (provided by Philips TL20W/01RS tubes; wavelength maximum 312 nm; spectrum shown in Cloix et al., 2012). At least 25 seedlings from each line were measured.

### Protein analysis

Wild‐type UVR8 and UVR8^C231S,C335S^ were expressed in *E. coli* and purified as detailed by Christie et al. (2012). Purified protein was exposed to 3 μmol m^−2^ sec^−1^ broadband UV‐B for 10 min. Non‐boiled protein samples were separated by a 10% SDS‐PAGE gel, which permits the resolution of UVR8 dimer and monomer (Rizzini et al., 2011). Gels were stained with Coomassie Blue.

Protein extraction from tissue samples, SDS‐PAGE, and immunodetection were undertaken as described previously (Kaiserli & Jenkins, 2007). The proteins were analysed by 10% SDS‐PAGE and transferred to nitrocellulose. Immunodetection on Western blots was with one of the following antibodies: anti‐UVR8‐C (Kaiserli & Jenkins, 2007), anti‐COP1 (Lian et al., 2011), anti‐RUP2 (Liao et al., 2020), or anti‐GFP (Takara Bio, San Jose, CA, USA). Rubisco large subunit, stained by Ponceau S, was used as a loading control on Western blots.

To induce dimer monomerization, 14‐day‐old plants were exposed to 3 μmol m^−2^ sec^−1^ broadband UV‐B for 30 min. To monitor re‐dimerization, the UV‐B‐treated plants were transferred to darkness at room temperature for the indicated times. Total plant proteins were extracted and protein samples were prepared for electrophoresis without boiling. Protein samples were resolved by 10% SDS‐PAGE and UVR8 protein was detected on Western blots by anti‐UVR8‐C antibody.

### Anthocyanin

The method for anthocyanin extraction and quantification was described previously (Huang et al., 2012). Seedlings were harvested and weighed quickly, then placed into microcentrifuge tubes containing 350 μl of 18% 1‐propanol, 1% HCl, and 81% sterile dH_2_O. The tubes were boiled at 95°C for 3 min and then incubated in darkness for at least 2 h at room temperature. After a brief centrifugation in a bench top microcentrifuge for 30 sec, 250 μl of the extract was transferred into a new tube containing 750 μl of extraction solution. The amount of anthocyanins in the final extract was measured by spectrophotometry at 535 and 630 nm. The values are reported as (A535‐2*A630)/g fresh weight.

### Co‐IP analyses

For Co‐IP analyses, plants were grown on agar plates and exposed to UV‐B as described above. Whole cell protein extracts were prepared as described by Kaiserli and Jenkins (2007). The Co‐IP assays were carried out using anti‐GFP microbeads (μMacs, 130‐091‐125; Miltenyi Biotec, Bergisch Gladbach, Germany) to immunoprecipitate GFP‐UVR8, and the presence of COP1 and RUP2 in the immunoprecipitates was examined as described previously (Cloix et al., 2012; Liao et al., 2020). The ‘input’ samples applied to the microbead columns and the immunoprecipitate eluates were analysed by SDS‐PAGE followed by Western blotting and immunodetection using the anti‐GFP, anti‐COP1, and anti‐RUP2 antibodies mentioned above.

### BiFC

The UVR8, UVR8^C231S,C335S^, COP1, and RUP2 fusions were cloned into the pBiFCt‐2in1‐NC vector containing the N‐ and C‐terminal regions of YFP by Gateway™ cloning technology (Thermo Fisher Scientific, Waltham, MA, USA) (Grefen & Blatt, 2012). The *Agrobacterium* suspension containing the required constructs was infiltrated into *N. benthamiana* leaves as described by Liao et al. (2019). After 2–3 days, plants were exposed to 3 μmol m^−2^ sec^−1^ broadband UV‐B for 1 h before taking images. Confocal images were collected by a Zeiss LSM510‐META confocal microscope with a 20×/0.75‐NA objective lens. YFP fluorescence was excited by 514 nm light and collected over 520–565 nm.

### Y2H assay

For the Y2H assay, *UVR8*, *UVR8* ^*C231S*^, *UVR8* ^*C335S*^, *UVR8* ^*C231S,C335S*^, and *UVR8* ^*C74S,C127S*^ were cloned into the pGBKT7 vector containing the Gal4 DNA‐binding domain. *COP1*, *RUP1*, and *RUP2* were cloned into the pGADT7 vector containing the Gal4 activation domain. Control fusions contained either mammalian P53 or T‐antigen (positive control), or no inserts (negative control). Plasmids containing an activation domain and a binding domain fused to the indicated proteins were co‐transformed into the yeast strain AH109. The yeast growth assay was applied on the non‐selective solid medium (SD/−Trp/−Leu) or selective solid medium (SD/−Trp/−Leu/–His) at 30°C for 3 days in darkness or under 0.1 μmol m^−2^ sec^−1^ narrowband UV‐B light.

### FRET experiments

The FRET experiments were undertaken as reported by Liao et al. (2019). The UVR8, UVR8^D96N,D107N^, and UVR8^W285F^ constructs in the pFRET‐NcCg‐Dest vector were described previously (Liao et al., 2019). The UVR8^C231S,C335S^ construct was produced by the same method, introducing a PCR product of UVR8^C231S,C335S^ into the vector by Gateway™ cloning technology (Thermo Fisher Scientific) (Liao et al., 2019). The construct was checked by restriction enzyme digestion and sequencing and transformed in *Agrobacterium* GV3101 for subsequent transient expression in tobacco.

Single colonies of *Agrobacterium* containing the required constructs were used to produce cultures for transient expression in *Nicotiana tabacum*, Four‐ to six‐week‐old plants were used for leaf infiltration as described previously (Liao et al., 2019). Plants were moved back to the growth chamber and the expression was observed after 2–3 days using a Leica TCP SP8 FRET‐FLIM confocal microscope (Liao et al., 2019). Where indicated, leaves were exposed to broadband UV‐B as described above. GFP fluorescence was excited by the 8% 488 nm laser and collected over 500–535 nm with 30% gain value. To detect the FRET signal, mCherry fluorescence was collected over 590–645 nm with 100% gain value. mCherry also was separately excited by the 8% 552 nm laser as a mCherry control. The FRET efficiency was calculated as described in the figure legends.

### Replication and statistical analysis

Experiments were repeated three times with independent biological replicates. Statistical analysis of the data was performed on GraphPad prism software using the method indicated in the figure legends.

## CONFLICT OF INTERESTS

The authors declare that they have no competing interests.

## Supporting information


**Figure S1.** Amino acid sequence alignment of the GWRHT motifs in UVR8 proteins from different plant species.

**Figure S2.** Yeast two‐hybrid assays of interactions involving single C231S and C335S mutants.

**Figure S3.** Negative controls for the BiFC assays shown in Figures 3c and 5a.

**Table S1.** Primers used in this study.

## Data Availability

All relevant data can be found within the manuscript and its supporting materials.
