# Supplementary material for: Cysteines have a role in conformation of the UVR8 photoreceptor
Source: Plant J. 2022 Jun 20;111(2):583–94. doi: 10.1111/tpj.15841 (PMC9546227; doi:10.1111/tpj.15841)
Supplement: Supplementary file 1 — Figure S1. Amino acid sequence alignment of the GWRHT motifs in UVR8 proteins from different plant species. [file TPJ-111-583-s002.pdf]

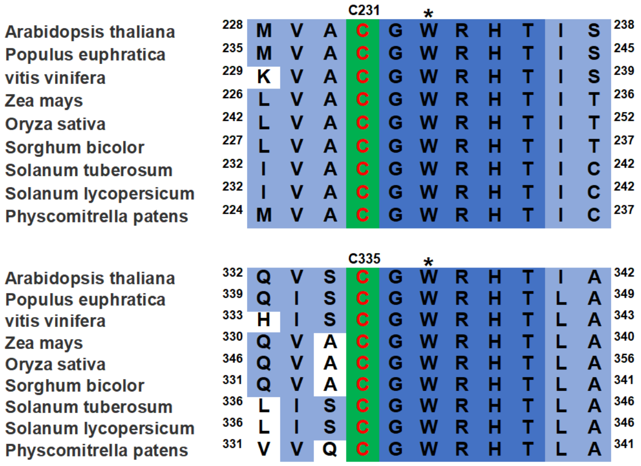

Figure S1: Amino acid sequence alignment of the GWRHT motifs in UVR8 proteins from different plant species.

The sequences were obtained from the NCBI protein database. The sequences of *Arabidopsis thaliana*, *Populus euphratica, Vitis vinifera*, *Zea mays*, *Oryza sativa*, *Sorghum bicolor, Solanum tuberosum*, *Solanum lycopersicum*, and *Physcomitrella patens* are shown. Amino acid sequence alignment showing the conservation of cysteine (marked by green box) in these two motifs. The asterisks marked tryptophan (W) are W233 and W337 in *Arabidopsis thaliana*, respectively.
