# Supplementary material for: Cysteines have a role in conformation of the UVR8 photoreceptor
Source: Plant J. 2022 Jun 20;111(2):583–94. doi: 10.1111/tpj.15841 (PMC9546227; doi:10.1111/tpj.15841)
Supplement: Supplementary file 2 — Figure S2. Yeast two‐hybrid assays of interactions involving single C231S and C335S mutants. [file TPJ-111-583-s003.pdf]

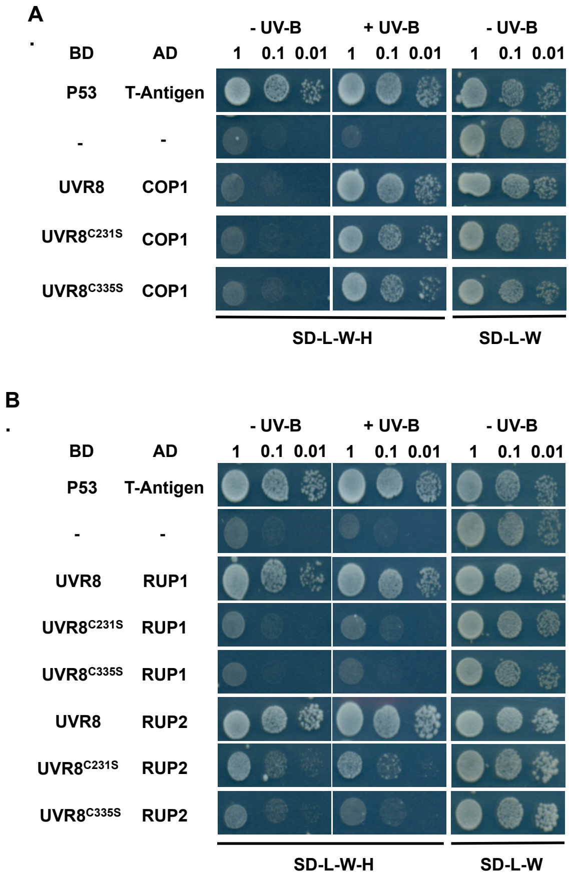

Figure S2: Yeast 2-hybrid assays of interactions involving single C231S and C335S mutants.

Yeast two-hybrid assays of COP1 **(A)** and RUP protein **(B)** interactions with wild-type UVR8, UVR8^{C231S} and UVR8^{C335S}. Suspended cells in serial dilutions (OD = 1, 0.1, 0.01) were spotted on selective medium (SD/-Leu/-Trp/-His, SD-L-W-H) or non-selective medium (SD/-Leu/-Trp, SD-L-W). Growth was assayed in the presence (+ UV-B) or absence (- UV-B) of UV-B. AD, activation domain; BD, binding domain. P53 and T-antigen were used as a positive control and empty vectors (-) as a negative control.
