# Supplementary material for: Cysteines have a role in conformation of the UVR8 photoreceptor
Source: Plant J. 2022 Jun 20;111(2):583–94. doi: 10.1111/tpj.15841 (PMC9546227; doi:10.1111/tpj.15841)
Supplement: Supplementary file 3 — Figure S3. Negative controls for the BiFC assays shown in Figures 3c and 5a. [file TPJ-111-583-s004.pdf]

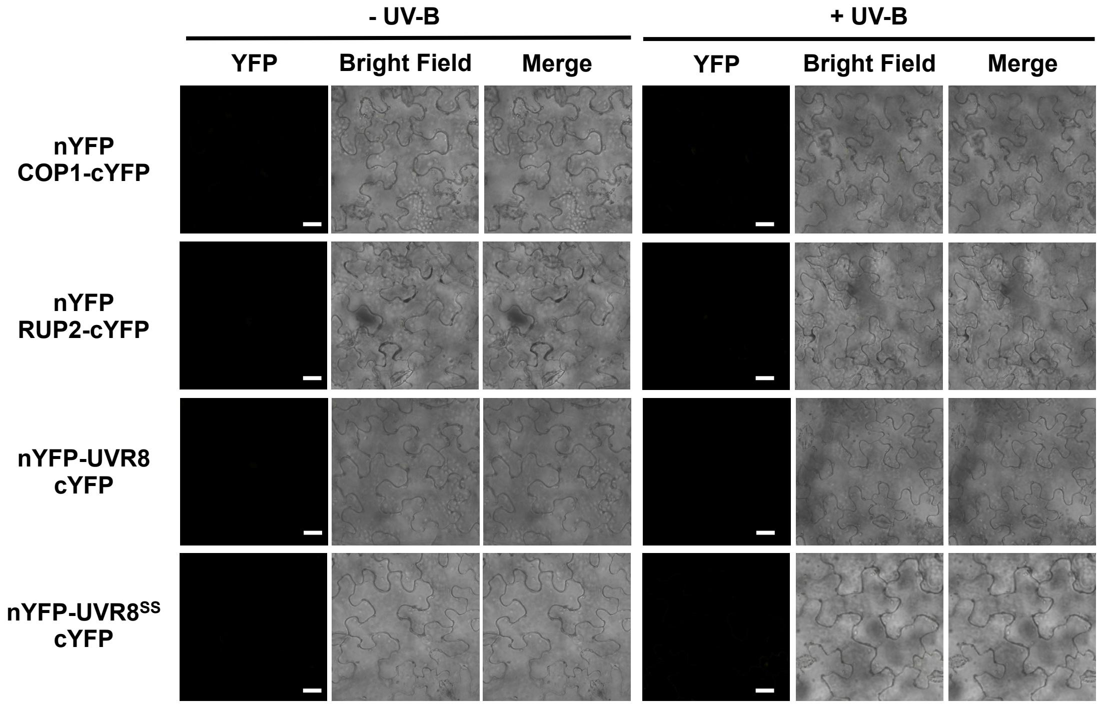

Figure S3: Negative controls for the BiFC assays shown in Figures 3C and 5A.

Plasmids expressing either N-terminal YFP or C-terminal YFP with the indicated fusions were infiltrated into *Nicotiana benthamiana* leaves. Plants were exposed (+ UV-B) or not (- UV-B) to 3 μmol m^{-2} s^{-1} broadband UV-B for 1 hour before taking images. Images from left to right are YFP fluorescence signal, bright field, and merged image. Bar = 20 μm.
