# Supplementary material for: Cysteines have a role in conformation of the UVR8 photoreceptor
Source: Plant J. 2022 Jun 20;111(2):583–94. doi: 10.1111/tpj.15841 (PMC9546227; doi:10.1111/tpj.15841)
Supplement: Supplementary file 4 — Table S1. Primers used in this study. [file TPJ-111-583-s001.pdf]

Primer Name	Primer Sequence
**Primers for Site-specific mutagenesis**	
C231S F	AATGTCAATGGTTGCTAGTGGATGGCGGCACAC
C231S R	GTGTGCCGCCATCCACTAGCAACCATTGACATT
C335S F	GAAAGTAGTTCAAGTCTCAAGTGGATGGAGACATACCTT
C335S R	AAGGTATGTCTCCATCCACTTGAGACTTGAACTACTTTC
C74S F	CAAATTGTTTCCGTTACCAGTGGTGCTGATCACACTG
C74S R	CAGTGTGATCAGCACCACTGGTAACGGAAACAATTTG
C127S F	GGATCAAGCAGATTGCTAGTGGGGATAGTCATTGT
C127S R	ACAATGACTATCCCCACTAGCAATCTGCTTGATCC
**Primers for plasmid construction**	
UVR8-EcoR1-F	TAGGCGAATTCGCGGAGGATATGGCTGCCGAC
UVR8-Sal1-R	GGCGTCGACTCAAATTCGTACACGCTTGAC
UVR8-Xho1-R	GGCGAGCTC TCAAATTCGTACACGCTTGAC
**Primers for Gateway Cloning**	
UVR8 F-attB1	GGGGACAAGTTTGTACAAAAAAGCAGGCTTAATGGCGGAGGATATGGCTGC
UVR8 F-attB3	GGGGACAACTTTGTATAATAAAGTTGTAATGGCGGAGGATATGGCTGC
UVR8 R-attB2-w/s	GGGGACCACTTTGTACAAGAAAGCTGGGTTCAAATTCGTACACGCTTGAC
UVR8 R-attB2-wo/s	GGGGACCACTTTGTACAAGAAAGCTGGGTGAATTCGTACACGCTTG
**Primers for qRT-PCR**	
HY5 for qPCR F	GCTGCAAGCTCTTTACCATC
HY5 for qPCR R	AGCATCTGGTTCTCGTTCTG
RUP2 for qPCR F	TGAATTCGATCCCACTGATAACA
RUP2 for qPCR R	AGGGAGGCCGTAAAAACGA
Actin2 for qPCR F	CTTACAATTTCCCGCTCTGC
Actin2 for qPCR R	GTTGGGATGAACCAGAAGGA

Table S1: Primers used in this study
